# Accuracy of radiographs in assessment of displacement in lateral humeral condyle fractures

**DOI:** 10.1007/s11832-014-0553-8

**Published:** 2014-02-02

**Authors:** Ashleen Knutsen, Tigran Avoian, Sean L. Borkowski, Edward Ebramzadeh, Lewis E. Zionts, Sophia N. Sangiorgio

**Affiliations:** 1 The J. Vernon Luck Research Center, Orthopaedic Institute for Children, 403 W. Adams Blvd., Los Angeles, CA 90007 USA; 2 UCLA/Orthopaedic Hospital Department of Orthopaedics, David Geffen School of Medicine at UCLA, 10833 Le Conte Ave 16-155 CHS, Los Angeles, CA 90095 USA

**Keywords:** Fracture displacement, Elbow fracture, Radiographic accuracy

## Abstract

**Purpose:**

Determining the magnitude of displacement in pediatric lateral humeral condyle fractures can be difficult. The purpose of this study was to (1) assess the effect of forearm rotation on true fracture displacement using a cadaver model and to (2) determine the accuracy of radiographic measurements of the fracture gap.

**Methods:**

A non-displaced fracture was created in three human cadaveric arms. The specimens were mounted on a custom apparatus allowing forearm rotation with the humerus fixed. First, the effect of pure rotation on fracture displacement was simulated by rotating the forearm from supination to pronation about the central axis of the forearm, to isolate the effects of muscle pull. Then, the clinical condition of obtaining a lateral oblique radiograph was simulated by rotating the forearm about the medial aspect of the forearm. Fracture displacements were measured using a motion-capture system (true-displacement) and clinical radiographs (apparent-displacement).

**Results:**

During pure rotation of the forearm, there were no significant differences in fracture displacement between supination and pronation, with changes in displacement of <1.0 mm. During rotation about the medial aspect of the forearm, there was a significant difference in true displacements between supination and pronation at the posterior edge (*p* < 0.05).

**Conclusion:**

Overall, true fracture displacement measurements were larger than apparent radiographic displacement measurements, with differences from 1.6 to 6.0 mm, suggesting that the current clinical methods may not be sensitive enough to detect a displacement of 2.0 mm, especially when positioning the upper extremity for an internal oblique lateral radiograph.

**Electronic supplementary material:**

The online version of this article (doi:10.1007/s11832-014-0553-8) contains supplementary material, which is available to authorized users.

## Introduction

Fracture of the lateral humeral condyle is the second most common elbow fracture in children [12, 17], reported to represent 12–20 % of pediatric elbow fractures [11, 19, 22] with an estimated annual incidence of 1.6 per 1,000 individuals [11]. Surgical treatment is recommended for fractures displaced more than 2 mm, either by closed reduction and percutaneous pinning [23] or open reduction and internal fixation [2, 4, 10, 11, 20, 24]. For non-displaced and minimally-displaced fractures, closed treatment using a long arm cast or splint is usually effective [3].

Determination of the magnitude of displacement can be difficult. Some authors have suggested ultrasound, magnetic resonance imaging (MRI), arthrography, or multi-detector computed tomography (CT) [5, 15, 18, 21, 27, 28]. Many of these methods involve greater expense, time, radiation exposure, painful examinations, or even sedation [26]. Therefore, initial displacement assessment is often determined using plain radiographs.

Several criteria have been recommended to predict the stability of minimally-displaced lateral condyle fractures using the standard radiographs [3]; however, despite adherence to these guidelines, fractures showing minimal displacement on initial radiographs may still displace further. Subsequent displacement while in a cast may lead to delayed union or non-union requiring operative treatment [2, 8, 9, 11, 13, 20].

Finnbogason and colleagues [8] used radiographic criteria to determine the stability of lateral condyle fractures that were non-displaced or minimally displaced, based on the appearance of the fracture line on an anteroposterior radiograph of the elbow. They classified fractures into three groups with the following criteria: (1) fracture in the metaphysis cannot be followed all the way to the epiphyseal cartilage; (2) fracture line can be observed to the epiphyseal cartilage; and (3) the fracture gap is as wide medially as laterally. The authors reported that all specimens in group 1 remained stable, but that approximately 20 % of group 2 and approximately 40 % of group 3 displaced in the cast.

Later, Song et al. [25] emphasized the importance of internal oblique radiographs for determining the stability of non-displaced and minimally displaced lateral condyle fractures. They reported that for 70 % of fractures the amount of displacement revealed on an anteroposterior radiograph differed substantially from that shown on an internal oblique radiograph. They also noted that for 75 %, the fracture patterns graded according to the Finnbogason criteria differed between the two views. These authors reasoned that because the plane of the fracture was often directed posterolaterally, the internal oblique radiograph brought the fracture line into better view, often changing the magnitude of the fracture gap that was present.

**Fig. 1:**
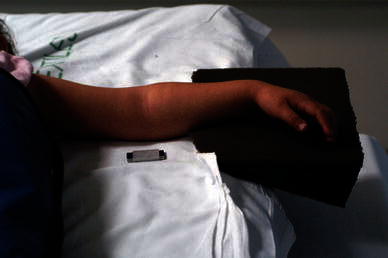
Internal oblique radiograph positioning: arm extended, forearm pronated, anterior surface of elbow at 45 degrees

Internal oblique radiographs are taken by pronating the forearm (Fig. 1a). Therefore, it is possible that positioning of the arm may lead to further displacement of the fracture, either by passive tightening or active contraction of the muscles attached to the fragment (including the extensor digitorum communis, extensor digiti quinti, extensor carpi ulnaris, and anconeus), or the fracture fragment may be further separated by forces generated across the distal humerus by the weight of the hand and forearm.

The purpose of this study was to (1) assess the effects of forearm rotation on the displacement of a simulated lateral humeral condyle fracture in a controlled biomechanical model, and to (2) determine the accuracy of radiographic measurements. Displacement measurements were obtained during supination, neutral position, and pronation while the forearm was rotated about the central axis of the forearm, isolating the effects of muscle pull and limiting varus bending. The same measurements were obtained in the same forearm positions about the medial aspect of the forearm as it was resting on the table, simulating positions and muscle tensions when obtaining an internal oblique radiograph. Fracture line displacements were measured by anteroposterior and lateral radiographs. The accuracy of displacement measurement was determined by comparing displacement measured from radiographs to displacement measured using an optical motion tracker.

## Materials and methods

### Specimen preparation

**Fig. 2:**
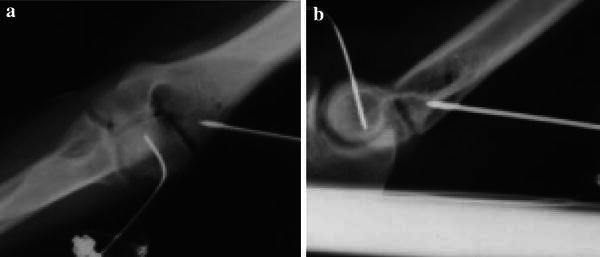
Anteroposterior (**a**) and lateral (**b**) radiographs of simulated lateral condyle fractures. Motion-capture flags, mounted on k-wires and attached to the proximal humerus and distal fracture fragment, are seen in the radiographs

Fresh-frozen adult human cadaveric arms were obtained from the International Institute for the Advancement of Medicine (Jessup, PA). Specimens were stored at −20° C and thawed to room temperature prior to the creation of fractures and experimentation. Two specimens were selected for pilot work and four were selected as experimental specimens. To create the fractures, an anterolateral approach was used. A Milch type II fracture was created, which extended into the apex of the trochlea [22], taking care to keep the lateral collateral ligament and the origin of all of the extensor muscles intact. A fracture fragment was created with a posterolateral metaphyseal portion to simulate the clinical situation (Fig. 2). Once the fracture creation technique was established and reproducibility of the fracture achieved, fractures were created in four experimental specimens.

### Apparatus

**Fig. 3:**
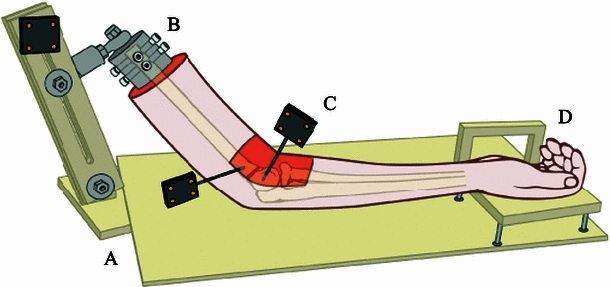
Schematic illustration of the simulated fracture specimen in the holding device: *A* plexiglass jig, *B* ball joint and steel pot to hold the proximal humerus, *C* motion-capture flags, *D* mini-table for hand to rest on

A custom apparatus was designed and fabricated to hold the arm in the desired positions during experimentation (Fig. 3). A plexiglass apparatus was attached to the proximal end of the humeral shaft of the specimen, and distally to the hand. A stainless steel pot held the proximal humerus with four pointed-tip screws, and prevented rotation or varus/valgus movement. A set screw was placed on the ball joint above the proximal humerus fixation pot. Once the arm was secured and the initial placement (i.e., non-displaced fracture) was verified, the set screw was tightened. Once the set screw was fastened, no varus/valgus motion or rotation of the humerus was allowed. The apparatus allowed for variable degrees of elbow flexion and extension. To enhance radiographic visibility of the fracture line, an opening was made at the base of the plate, inferior to the elbow.

In order to eliminate the influence of the arm’s natural weight on displacement, a plexiglass frame was constructed to stand on top of the existing elbow apparatus. The variable height table allowed the arm to rest in place, similar to the position of the extremity when taking a clinical radiograph. Another support was constructed to hold the hand slightly superior to the elbow. It had two removable walls, to secure the hand on the medial and lateral edges.

### Radiographic measurements

Standard anteroposterior and lateral radiographs were taken of the distal humerus. For anteroposterior radiographs, care was taken to position the medial and lateral humeral condyles parallel to the X-ray cassette. The lateral radiographs were obtained and deemed acceptible if they showed that the condyles were superimposed on the image, indicating a true lateral view.

### Motion capture measurements

**Fig. 4:**
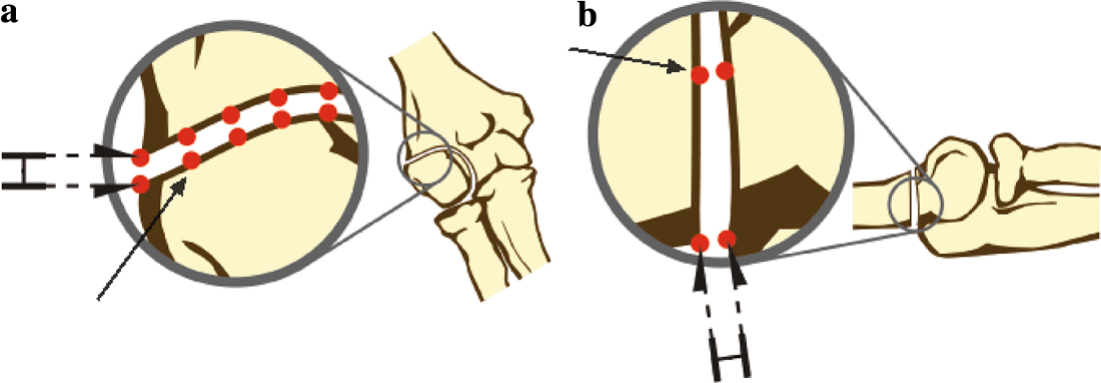
Illustration of points used to measure true displacement (*arrows*) using the motion-capture system and radiographic locations used to measure apparent displacement (*double arrows*) of the lateral edge of the fracture site (**a**) and of the posterior edge of the fracture site (**b**)

An Optotrak 3020 Motion Capture System (Northern Digital Inc, Waterloo, Ontario, Canada) was used to measure 3D translations and rotations of the fractured fragment relative to the body of the humerus, throughout experimentation. This motion tracking system has an accuracy of 0.1 mm and a resolution of 0.01 mm at a distance of 2.25 m [6, 7, 14, 16]. Two LED-motion flags were attached to each specimen, one to the proximal humerus and the other to the distal fractured fragment (Fig. 3). Seven points on each side of the fracture, that is, on both the proximal humerus and distal fragments, were digitized to determine critical fracture surface positions in space (Fig. 4). Motion was sampled throughout position changes of the forearm.

### Experimentation

#### Rotation about the central axis of the forearm (pure rotation)

Following fracture creation, each specimen was mounted in the apparatus, fixed at the proximal humerus and at the hand, and instrumented with motion-tracker flags. The specimen was initially positioned at 0° rotation (full supination) and at a degree of flexion/extension of the elbow such that the fracture was not displaced. The specimen was sequentially rotated 90° from the initial supine position (0°) to fully pronated (180°), such that the central axis of the forearm remained fixed (pure rotation). Fracture displacement was measure in three positions: 0° (supination), 90°, and 180° (pronation). Two radiographs (anteroposterior and lateral) were taken in each position for a total of six radiographs. 3D displacements between fractured surfaces were recorded throughout testing.

#### Rotation about the medial aspect of the forearm (hinged rotation)

Each specimen was again mounted in the apparatus and fixed at the humeral head; however, in this experiment, the hand was not constrained. No further preparation was necessary, as each specimen was already instrumented during the pure rotation measurements. Again, the specimen was initially positioned at 0° rotation (supination) and at a degree of flexion/extension of the elbow such that the fracture was not displaced. The forearm was then pronated 180° by rotating the forearm in the coronal plane along the medial aspect of the forearm. Again, standard anteroposterior and lateral radiographs were taken with the arm in each position (0° and 180°) and 3D distances were recorded throughout testing.

### Data acquisition and measurements

The displacement of the fracture fragment apparent on all radiographs was assessed by two independent orthopedic surgeon observers. From the anteroposterior radiographs, displacement was assessed at the lateral edge of the fracture. Using the lateral radiographs, the displacement was assessed at the posterior edge of the fracture. These measurements were considered to be the apparent fracture displacements (Fig. 4).

Using the motion-capture system, true real-time measurements of the displacement were recorded. Each of the corresponding digitized points, which are the seven pairs of digitizations representing a point on each the fracture fragment and the proximal humerus, were measured in 3D space throughout the experiments. The distances were recorded using NDI First Principles software (Northern Digital Inc, Waterloo, Ontario, Canada). The two distances along the fracture line focused on in this study were the displacement at the lateral edge (proximal humerus lateral edge to fractured fragment lateral edge) and the posterior edge (proximal humerus posterior edge to fractured fragment posterior edge) (Fig. 4). These measurements were considered to be the true fracture displacements.

### Statistical analysis

The categorical input variables for this study were the method of rotation (pure, hinged), and degree of rotation: 0° (supine), 90° (neutral), and 180° (pronated). The primary outcome variables were (1) true fracture displacement at lateral and posterior edges, and (2) apparent fracture displacement at the lateral and posterior edges. The true and apparent displacements were compared as a function of method of rotation (pure or hinged)) and degree of rotation. SPSS 15.0 statistical analysis software (SPSS, Inc., Chicago, IL) was used to perform paired-samples *t* tests to evaluate the main objectives of the study: (1) the effect of positioning on fracture motion, and (2) the difference between true fracture-displacement measurements and apparent fracture-displacement measurements. The associated *p* values were used to determine statistical certainty.

## Results

### Fracture creation

Lateral humeral condyle fractures were successfully created in three of the four specimens. In one specimen, the fracture line extended too far medially, and was not representative of a true lateral condyle fracture, and thus was removed from the analysis. The analysis was performed on the *n* = 3 remaining specimens.

### Fracture displacement

#### Pure rotation

During pure rotation from supination to pronation, there was minimal change (<1 mm) in true or apparent fracture displacement (Tables 1, 2). However, the true fracture-displacement measurements showed a statistically significant change in fracture displacement at the lateral edge when rotating from supination to 90° (*p* = 0.047). On average, at the lateral edge, the true fracture displacement remained constant through rotation from supination to pronation, with maximum displacement at 90°, while the apparent fracture displacement slightly decreased (1.6–1.2 mm). Measurements at the posterior edge showed inconsistent results. Both the true and apparent fracture displacements slightly increased when rotating from supination to pronation.

**Table 1:** Lateral edge measurements

	True displacement (by motion tracker) (mm)	Apparent displacement (by radiograph) (mm)	*P* value
‘Pure’ supination	3.5 ± 2.0	1.6 ± 0.5	0.16
‘Pure’ 90° (neutral)	4.0 ± 2.1	1.7 ± 0.7	0.10
‘Pure’ pronation	3.5 ± 2.3	1.2 ± 0.6	0.14
‘Hinged’ supination	4.4 ± 2.1	2.2 ± 1.2	0.08
‘Hinged’ pronation	3.7 ± 2.7	2.1 ± 2.2	0.03

Values reported as mean ± standard deviation. *P* value from paired-samples *t* test comparing true and apparent displacements

**Table 2:** Posterior edge measurements

	True displacement (by motion tracker) (mm)	Apparent displacement (by radiograph) (mm)	*P* value
‘Pure’ supination	5.9 ± 4.3	1.6 ± 0.9	0.22
‘Pure’ 90° (neutral)	6.6 ± 4.9	1.6 ± 1.2	0.20
‘Pure’ pronation	6.4 ± 5.0	1.9 ± 1.2	0.27
‘Hinged’ supination	6.1 ± 5.3	1.7 ± 1.1	0.30
‘Hinged’ pronation	7.9 ± 6.1	1.9 ± 1.0	0.24

Values reported as mean ± standard deviation. *P* value from paired-samples *t* test comparing true and apparent displacements

#### Hinged rotation

On average, the fracture displacement at the lateral edge decreased slightly when rotating from supination to pronation (Table 1). In contrast, the mean fracture displacement at the posterior edge increased (Table 2). While in both cases, the change in displacement observed from the apparent fracture displacement measurements was small, the mean true fracture displacements were 0.7 mm lower at the lateral edge and 1.8 mm higher at the posterior edge (*p* = 0.054).

### Accuracy in apparent fracture displacement measurements

The pure rotation experiments showed that, on average, the true fracture displacement measurements were larger than the apparent fracture displacement measurements, with a difference between the two ranging from 1.9 to 5 mm. However, when the true displacements were compared to apparent displacements, using paired analysis, no consistent differences were found. (Tables 1, 2). The hinged rotation experiments showed a similar trend, with the difference between true and apparent fracture displacement measurements ranging from 1.6 to 6.0 mm. However, this difference was found to be statistically significant only in the pronated position at the lateral edge of the fracture (*p* = 0.032). On average, the standard deviations in true fracture-displacement measurements were higher than apparent fracture-displacement measurements at both edges.

## Discussion

We evaluated the effect of rotation around the central axis of the forearm (pure rotation), and rotation around the medial edge of the forearm (hinged rotation) on displacement of a simulated lateral humeral condyle fracture using an adult cadaver model. Rotation around the central axis of the forearm isolated the effect of pull of the lateral collateral ligament and the muscles attached to the fracture fragment, while rotation around the medial edge of the forearm simulated positioning of the extremity used in the emergency room to obtain a lateral oblique radiograph.

We observed little change and no significant differences in fracture displacement with rotation of the forearm about the central axis; however, during the rotation about the medial aspect of the forearm, there was a significant difference in true fracture-displacement measurements between supination and pronation at the posterior edge (*p* < 0.05).

Overall, true displacement measurements were larger than apparent displacement measurements, with differences ranging from 1.6 to 6.0 mm. This finding supports a clinical study by Badelon et al. [2], who reported that fracture displacement found at surgery is often underestimated on radiographs. This finding is important since surgical treatment of this fracture is recommended for fractures displaced more than 2.0 mm [2, 4, 10, 20, 23, 24].

In order to obtain an external oblique radiograph of the elbow, the forearm is pronated (Fig. 1). Arnold et al. [1] performed a cadaver study reporting that pronating the forearm caused the brachioradialis-extensor muscle group to become taut. We speculated that pronating the forearm when obtaining a radiograph may affect the displacement of the fracture, through either increased tension through the anatomic structures that insert on the lateral condyle or by producing a varus moment at the fracture site; however, our findings did not support this, perhaps due to an insufficient number of experimental specimens.

The current study had several limitations that should be considered. First, sample size (*N* = 3) was small, and thus we may not have represented all of the different lateral condyle fracture patterns that are seen in the general population. That is, the study was limited to one type of fracture pattern, which may not thoroughly represent the clinical aspects of displacement as a function of elbow motion represented in the present study. Moreover, there is large variation among cadaveric specimens in general, and the specific anatomy and geometry of the elbow in these specimens may have affected the results as well. Second, adult cadaveric specimens were used, as pediatric cadaveric specimens are unavailable in the United States for biomechanics research. Furthermore, to maintain consistency throughout testing, the forearms were rotated about two axes: the central axis of the forearm (isolating the effect of muscle pull), and the lateral edge of the hand (inducing varus bending). Despite showing minimal differences during these rotations, the experimental setup may have overly limited the forces. When a patient pronates his arm, the medial aspect of the forearm does not remain fixed, and the result is a hinged pronation with a much greater arc length. As a result, higher varus bending may be seen clinically, resulting in larger forces, thus affecting fracture displacement more so than shown in this study.

In conclusion, the static pull of the muscle during pure rotation did not cause significant changes in fracture displacement; however, simulating conditions experienced clinically during an internal oblique radiograph resulted in a significant difference in fracture displacement. Additionally, radiographic measurements of fracture displacement were smaller than true displacement measurements, suggesting that current clinical methods may not be sensitive enough to detect the commonly used maximum displacement of 2.0 mm, particularly when positioning the upper extremity for an internal oblique lateral radiograph.

## Electronic supplementary material

Below is the link to the electronic supplementary material.

Supplementary material 1 (DOCX 828 kb)
