# Supplementary material for: Accuracy of radiographs in assessment of displacement in lateral humeral condyle fractures
Source: J Child Orthop. 2014 Feb 2;8(1):83–9. doi: 10.1007/s11832-014-0553-8 (PMC3935020; doi:10.1007/s11832-014-0553-8)
Supplement: Supplementary file 1 — Supplementary material 1 (DOCX 828 kb) [file 11832_2014_553_MOESM1_ESM.docx]

Prof. Fritz Hefti
Editor-in-Chief
Journal of Children's Orthopaedics

November 13, 2013

Dear Prof. Hefti,

My colleagues and I are pleased to send you our revised manuscript entitled “Accuracy of Radiographs in Assessment of Displacement in Lateral Humeral Condyle Fractures.” We appreciate the input from the reviewers and have incorporated all of their suggestions and comments in our revised manuscript. A detailed response of how each concern was addressed is included in the attached “Response to Reviewers.” In addition to these changes, we went through the manuscript and made several small changes to improve the flow, correct the grammar, or correct typos. All specific changes made in response to reviewer comments, or significant changes to the text have been BOLDED in the revised manuscript. We thank the reviewers for their comments and questions and we hope that our changes have addressed all of their previous concerns.

Sincerely,

Ashleen Knutsen, M.S.
